# When You are About to Diagnose Chronic Hemolytic Uremic Syndrome, Please Think More Deep

**DOI:** 10.4061/2010/285952

**Published:** 2010-10-25

**Authors:** Doaa Mohammed Youssef, Doaa Mostafa Tawfeek

**Affiliations:** Pediatric Department, Zagazig University, Zagazig 44519, Egypt

## Abstract

Antiphospholipid syndrome is one type of immunological diseases which may be primary or secondary characterized by repeated thrombosis that it may be called “sticky blood syndrome”. Although a well-known disease in gynecology, there is no sufficient data in pediatrics field; so we see that it is important to discuss this interesting case.

## 1. Introduction

Antiphospholipd syndrome is a disorder of recurrent vascular thrombosis, pregnancy loss, and thrombocytopenia associated with persistently raised levels of antiphospholipid antibodies [1]. It may be primary, occurs alone or secondary, associated with other autoimmune or rheumatic diseases, also known as Hughes syndrome, as described by Dr. Graham Hughes in 1983. In recent years, the features of antiphospholipid syndrome have been increasingly recognized in children [2].

## 2. Objective

We discuss a case with different presentation. It was diagnosed at first as hemolytic uremic syndrome and passed through different other presentations which fulfilled the diagnostic criteria of antiphospholipid syndrome.

## 3. Case Presentation

The case was a female female patient 13 years old, 1st kid of a low socioeconomic state family, from Zagazig. The condition started (May 2007) by acute onset of vomiting not responding to any medication followed on 2nd day by vaginal bleeding for 13 days. She was referred to insurance hospital where they discovered on clinical examination, jaundice and pallor too. The patient was then referred to our hematology unit where complaining of dark colored urine, puffy eyelid, and on examination there was purpura on abdominal, and investigation at that time revealed the following.

CBC: Hb 5.7 gm\dL, RC 21%, platelet 28000, serum Creatinine 2 mg\dL, blood Urea 113 mg\dL, Coombs test both direct and indirect were negative, Total billruibin 2.2 mg\dL, direct 0.2 mg\dL, ESR 75, Urine analysis showed RBC's over 100. 

Our differential diagnosis at that level was as follows:

1. hemolytic uremic syndrome,
2. autoimmune hemolytic anemia,
3. immunological disease, for example, SLE,
4. TTP (thrombotic thrombocytopenic purpura),
5. chronic hemolytic anemia on exacerbation,
6. post infection, for example, HBV.

So we performed next investigations: blood film showed fragmented red cells, Coombs test again direct and indirect were negative, Osmotic fragility was negative, Hemoglobin electrophoresis was normal, ANA negative, Anti-double-strand DNA negative, Abdominal US enlarged kidneys with mild splenomegaly, Hepatitis markers HBV weak positive (HBS Ag) by PCR negative, C3 normal. So diagnosis of hemolytic uremic syndrome was the most likely diagnosis at that level with raising Creatinine to 10.5 mg\dL, dialysis was initiated (via subclavian catheter), plus supportive blood and platelet transfusion and Electrolytes support.

Actually our patient showed slow response to dialysis that she was dialysis to the extend dependent for 7 weeks; GFR by DTPA at that time was 15 mL\mint\1.73 m. So AVF was done.

Apart from 3 admissions to ICU with malignant hypertension, no other systems were affected in the next 7 weeks. Blood pressure was difficult to control because Dina was on 4 antihypertensives. 

So it is a type of resistant HUS. We gave plasma transfusion repeatedly to cut off the circuit of pathogenesis; actually it was a great surprise that Dina began to be off dialysis, with normal CBC. 

The patient was discharged on conservative management of CRF and a close followup twice weekly was done. Investigations on follow up after 2 months of admission (July 2007) showed CBC RC 3%, Hb 9 gm\dL, Platelet 224,000, Urea 42 mg\dL, and Creatinine 1.3 mg\dL. 

The patient readmitted 3 weeks later with acute pallor and jaundice again with purpura and hypertension with palpitation.

Investigations at that time (August 2007) showed the following results: RC 7.8%, Hb 7.6 gm\dL, Platelet 94,000, Coombs negative, Creatinine 4.9 mg\dL, Urea 167 mg\dL, Urine RBCS over 100, C3 consumed (0.5), ANA and Anti-double-strand DNA were negative, ANCA negative, ESR 85 1st hour, 126 2nd hour. Bone marrow biopsy was done and revealed hyperactive bone marrow with no abnormal or megakaryocytic changes. ECG showed sinus tachycardia. echocardiography showed grade 3\4 miteral regurgitation, grade 1\4 Aortic regurgitation, pulmonary hypertension, and moderate pericardial effusion. 

Plasmapheresis was initiated 4 sessions at one week with no improvement so redialysis was repeated.

One week later the patient complained of abdominal pain, abdominal US, right kidney enlargement for Doppler study, and right renal artery blunted systolic filling denote obstruction at subsegmental branching.

 Renal biopsy was done revealing, Microangiopathic glomerulonephritis HUS versus other vasculitis. 

Our differential diagnosis at this level was:

1. SLE,
2. other types of vasculitis,
3. 2ry antiphospholipid syndrome,
4. TTP,
5. other cause (protein S.C deficiency),
6. 1ry antiphospholipid syndrome.

Anticardiolipin was done and it was positive.

So diagnosis of antiphospholipid syndrome was done and treatment was initiated as follows: steroid was initiated in September 2007. Anticoagulation heparin followed by low molecular weight heparin was given as well as antiplatelet, Cytotoxic in the form of cyclophosphamide 2 pulses I.V. monthly apart. 

After two weeks Hemolysis regressed, but renal function was the same, GFR 30 mL\mint\1.73 m (improved). But the patient developed new signs of vascular affection in the form of Lived reticularis on abdominal wall and leg with repeated chest pain.

Note that the patient was on regular dialysis through AVF, blood pressure fluctuations were on the high level always (so stoppage of anticoagulant was done), and antidepressant was added; blood pressure was controlled by it plus using one antihypertensive only but the patient lost 9 kg in the next 6 months. 

She complained of recurrent massive pericardial effusions and responded to daily dialysis for 15 days each time for 4 times with stoppage of anticoagulant. 

Intravenous immunoglobulin was given 5 days a week for three weeks, but unfortunately with no response as regards hemolysis or C3 level. 

Reinvestigating for ACL, ANA, Anti-douple-strand DNA, ANCA, and all four tests were negative. And two weeks later repeated ACL was done and was negative. 

The patient was on the same management with gradual decrease of steroid because the only response was raising C 3 level without clinical response for 3 months; on the other hand, hypocalcaemia and other signs of side effects began. 

In November 2007, AVF thrombosis proved by Doppler recanalisation was done. 

 In December 2007, echocardiography was done for repeated chest pain with normal ECG showing unequal septal movement evident of myocardial infarction. Unfortunately, cardiac enzymes were not available at that time. 

In January 2008, sever chest infection followed by hemoptysis occurred. Tuberculin test done with double dose was negative and CT chest revealed wedge pulmonary infarction. 

In February 2008, head nodding and static tremors, possibility of Parkinson's (basal ganglia vascular affection) occured, but the result of MRI came to be frontal lobe atrophy from repeated infarctions mostly. 

In March 2008, AVF rethrombosis occured and it was documented by Doppler but after 24 hours of detection of thrombosis, fistula leakage and emergency closure were done. 

In April 2008, the patient developed sever chest pain diagnosed as pulmonary infarction for which she was admitted to intensive care unit and unfortunately died. 

## 4. Discussion

The description of antiphospholipid syndrome has been one of the most striking developments in the field of immunology in the last two decades [1] the earliest descriptions of the association between a circulating anticoagulant and vascular thrombosis in pediatric population are those of Olive et al. 1979 [3]. 

Our case had the following positive clinical data: hemolytic anemia, thrombocytopenia, hematuria with impaired kidney function, interarenal vascular occlusion, pulmonary infarction, cardiac infarction, valvular lesion, recurrent pericardial effusion, AVF thrombosis, and neurological insult. 

And the case had the positive laboratory data as follows: ACL positive once, ANA, Anti-double-stranded DNA negative, ANCA negative, C3 consumed, documented thrombosis (interarenal, intraglomular, pulmonary, AVF, cardiac, CNS), and normal PT, PTT. 

For diagnosis of antiphospholipid syndrome, the patient should fulfill the diagnostic preliminary criteria by Sapporo which was proposed in 1998 one clinical plus one lab [1, 4].

(a) Clinical criteria are as follows:
  (1) vascular occlusion artery, vein or minute vessels in tissue or organ with no inflammatory reaction around,
  (2) pregnancy related.
(b) Laboratory criteria were as follows:
  (1) ACL AB IgG or IgM positive in two separate samples 6 weeks apart,
  (2) lupus anticoagulant AB positive in two separate samples 6 weeks apart.

All criteria need not to be met to make a diagnosis of APS. 

Catastrophic antiphospholipids syndrome (CAPS) (Accelerated form of APS with consequent multiorgan failure, disturbance at small vessel level) hasinternational criteria for classification such as

(1) clinical evidence of vessel occlusions affecting 3 or more organs or systems,
(2) development of the manifestations simultaneously or in less than a week,
(3) confirmation by histopathology of small vessel occlusion in at least one organ,
(4) serological confirmation of the presence of APL (LA and/or ACL).

N.B. The kidney is the organ most commonly affected [5]. 

In our case the patient fulfilled criteria of a catastrophic antiphospholipid syndrome as the repeated ACL was negative, but our patient showed multiorgan failure, and there is small vessel affection as those appearing in renal biopsy (Microangiopathic glemuleropathy). The main organ affected and one of the earliest organ affections was the kidney, and our case died after 11 months of diagnosis although we start all kinds of therapy of APL. 

Our case in clinical presentation showed veins affection (Lived reticularis), arterial affection (renal thrombotic Microangiopathic, myocardial infarction, pulmonary infarction, and brain ischemic manifestation), and also showed cardiac valve affection which was also described in APL [1]. 

Our patient 1st presentation was hemolytic anemia and thrombocytopenia which made us think that it is a case of hemolytic uremic syndrome, but it was actually a spectrum of clinical presentation of APL [2]. 

As regards differential diagnosis of our case, the main differential diagnosis was to exclude SLE as recently the presence of APL in lupus patients has been associated with development of occurrence of neuropsychiatry manifestation [6] and ischemic Microangiopathic nephropathy [7], as many studies reported prevalence of ACL and LA in juvenile SLE ranges from 19%–87% and from 10%–62% [1], also the possibility of transformation of 1ry APL to SLE after 6 months [1]. So we repeatedly sample our patient for ANA Anti-double-stranded DNA and it was negative all through till her death raising the possibility of 1ry APL rather than 2ry APL of SLE but this didnnot exclude the possibility of lupus-like syndrome. 

As regards management of APL, being symptomatic APL, that is, APL positive patient with thrombosis, it needs the following:

(1) anticoagulant like other cases with thrombosis, although the duration and intensity of antithrombotic therapy are not yet clearly established [8]. We started low molecular weight heparin followed by warfarin, but we needed to stop this treatment for many times because of uncontrolled hypertension or pericardial effusion;
(2) high doses of corticosteroid, cyclophosphamide, Intravinous immunoglobulin, and Plasmapheresis as well as hemodialysis all were tried as all are recommended in life-threatening APL or catastrophic antiphospholipid [9];
(3) although we gave many modalities of treatment, we lost our patient in a severe attach of pulmonary infarction and this showed us the important and the aggressive form of this disease, as high mortality—up to 48% despite antithrombotic therapy—had been reported in many studies [1, 5, 9].

## 5. Conclusion

APL is recognized increasingly as a leading cause of vascular thrombosis in pediatric population; also catastrophic antiphospholipid syndrome should take much care and recognition by pediatricians being one of severe and rabidly fatal disease; we saw to share this case and we suggest further studies about diagnosis, presentation, and management of this serious disease.

## Figures and Tables

**Figure 1:**
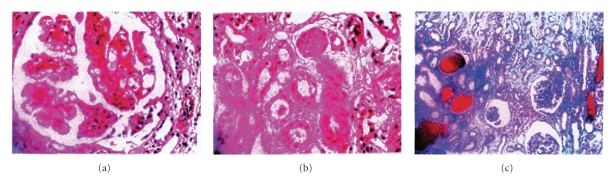
Renal biopsy showing Microangiopathic glomerulonephritis.

**Figure 2:**
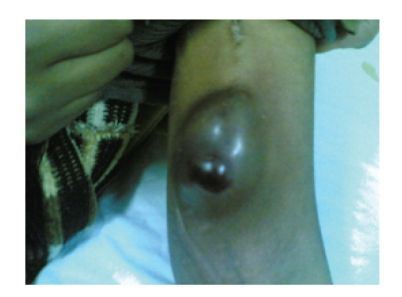
AVF rethrombosis and impending rupture.
